# Survival Determinants in Temporal Bone Squamous Cell Carcinoma: A Systematic Review and Meta-Analysis

**DOI:** 10.3390/jcm15187143

**Published:** 2026-09-15

**Authors:** Martina Sebastiani, Alessandro Facheris, Daniele Bernardeschi, Luca Canali, Giovanni Colombo, Matteo Di Bari

**Affiliations:** 1 Department of Biomedical Sciences, Humanitas University, 20072 Pieve Emanuele, MI, Italy; martina.sebastiani@humanitas.it (M.S.);; 2 Otorhinolaryngology Unit, IRCCS Humanitas Research Hospital, 20089 Rozzano, MI, Italy; luca.canali@humanitas.it; 3 Service d’Oto-Rhino-Laryngologie, Hôpital Pitié-Salpêtrière, AP-HP, Sorbonne Université, 75013 Paris, France; daniele.bernardeschi@aphp.fr; 4 Otorhinolaryngology, Head and Neck Department, Ospedale Nuovo di Legnano, ASST Ovest Milanese, Via Papa Giovanni Paolo II, 20025 Legnano, MI, Italy; mattediba@gmail.com

**Keywords:** squamous cell carcinoma, external ear canal, Pittsburgh classification

## Abstract

**Background/Objectives**: Temporal bone squamous cell carcinoma (TBSCC) is a rare and aggressive malignancy with limited evidence regarding prognostic factors. This systematic review and meta-analysis evaluated the prognostic significance of tumor stage, cervical nodal status, and surgical margin status in patients with TBSCC. **Methods**: A systematic search of PubMed and Scopus was conducted from database inception to December 29, 2025, in accordance with the PRISMA 2020 statement. Studies reporting hazard ratios (HRs) for overall survival in patients with TBSCC staged according to the modified Pittsburgh classification were included. Pooled HRs were calculated using random-effects models. **Results**: Thirteen retrospective cohort studies including 801 patients were analyzed. Positive surgical margins were associated with the greatest increase in mortality risk (HR, 4.79, 95% CI, 2.35–9.75; *p* < 0.001), followed by cervical nodal metastasis (HR, 2.81; 95% CI, 1.72–4.60; *p* < 0.001) and advanced tumor stage (T3–T4 vs. T1–T2; HR, 2.39; 95% CI, 1.24–4.60; *p* = 0.009). Sensitivity analyses confirmed the robustness of the pooled estimates. **Conclusions**: Positive surgical margins, advanced tumor stage, and cervical nodal metastasis were all significantly associated with poorer survival, with positive surgical margins showing the largest pooled association with mortality among the three evaluated factors. Prospective multicenter studies are needed to validate these findings and improve prognostic stratification in this rare malignancy.

## 1. Introduction

Temporal bone squamous cell carcinoma (TBSCC) is a rare and aggressive malignancy most commonly arising from the external auditory canal (EAC), accounting for less than 0.2% of all head and neck cancers, with an estimated annual incidence of 1–6 cases per million population [1]. The term TBSCC is primarily an anatomical and historical designation, as most tumors arise from the squamous epithelium lining the EAC and subsequently extend into the temporal bone rather than originating from osseous tissue itself [2]. Accordingly, external auditory canal squamous cell carcinoma may represent a more anatomically precise description of the primary tumor site, although the term TBSCC remains widely used in the literature. Owing to its rarity and the nonspecific nature of early symptoms, including otorrhea, otalgia, and hearing loss, diagnosis is frequently delayed, and many patients present with locally advanced disease at the time of treatment [2].

Because of its low incidence, high-quality evidence guiding the management of TBSCC remains limited. Nevertheless, the modified Pittsburgh classification remains the most widely adopted staging system for clinical assessment, treatment planning, and prognostic stratification [3]. Surgical resection with curative intent represents the cornerstone of treatment for resectable disease and is commonly combined with postoperative radiotherapy in patients with advanced-stage tumors or adverse pathological features. Nevertheless, survival remains highly variable, ranging from excellent outcomes in early-stage disease to poor long-term survival in patients with locally advanced tumors [4,5].

Several clinicopathological factors have been proposed as determinants of prognosis in TBSCC, including tumor stage, cervical nodal involvement, facial nerve paralysis, patterns of tumor spread, and surgical margin status [5]. Among these, advanced tumor stage is the most consistently reported predictor of poor survival [6]. However, the prognostic significance of other variables, particularly cervical node metastasis and surgical margin status, remains less clearly established, with heterogeneous findings reported across published series [7].

The aim of this systematic review and meta-analysis was to quantitatively evaluate the prognostic impact of tumor stage, nodal status, and surgical margin status on overall survival in patients with TBSCC staged according to the modified Pittsburgh classification. Although previous systematic reviews have summarized survival outcomes and prognostic factors in TBSCC, the relative prognostic impact of these clinicopathological variables has not been quantitatively synthesized using pooled hazard ratios. Given the rarity of TBSCC, individual studies are frequently underpowered and report heterogeneous findings, highlighting the need for a quantitative synthesis of effect estimates. By pooling hazard ratios across the available literature, we sought to provide more robust estimates of the relative prognostic importance of these factors and to support clinical decision-making and risk stratification.

## 2. Materials and Methods

### 2.1. Search Strategy

**Figure 1:**
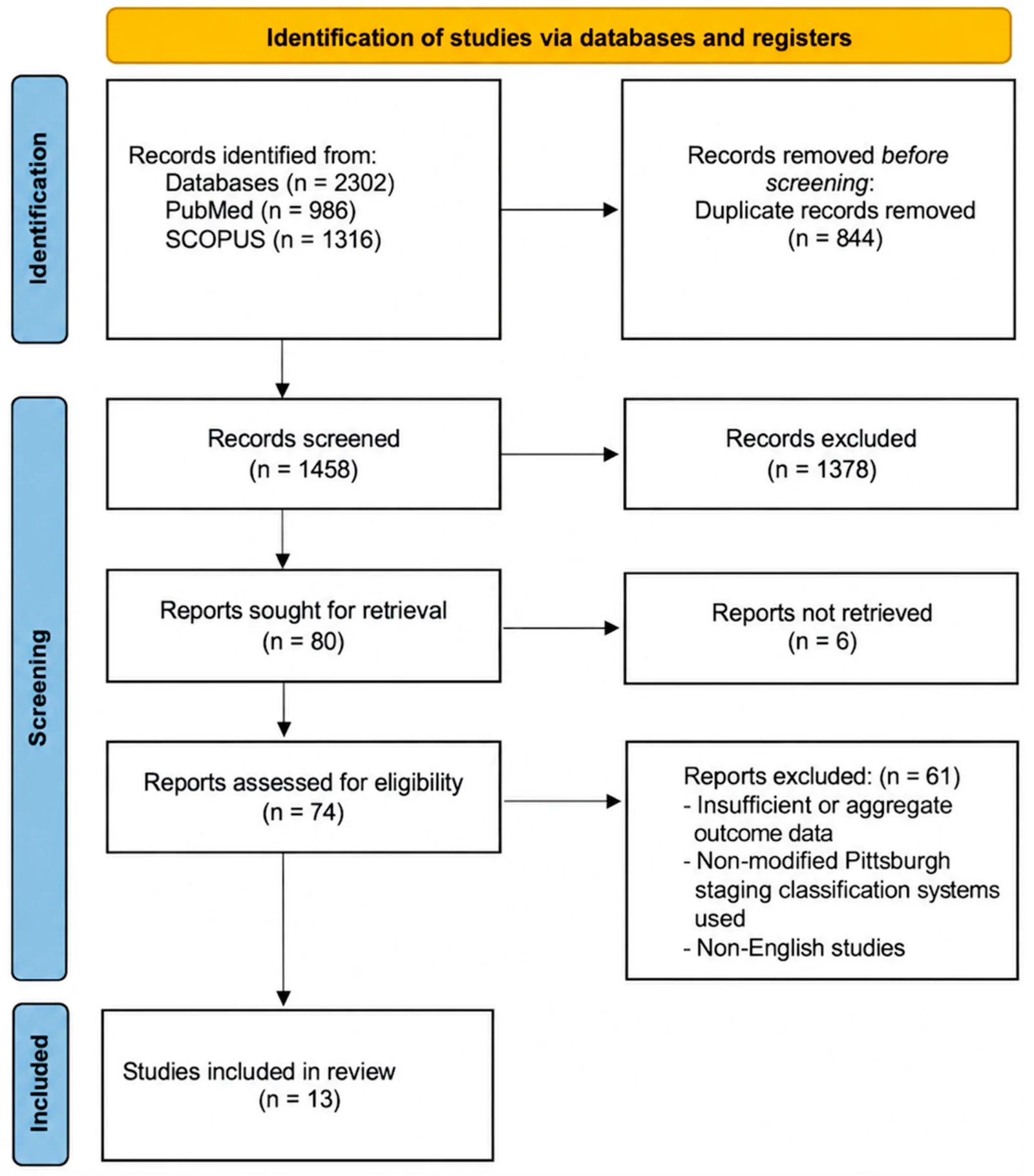
PRISMA diagram of the study.

This systematic literature review and meta-analysis was conducted in accordance with the Preferred Reporting Items for Systematic Reviews and Meta-Analyses (PRISMA) 2020 statement [8] (Figure 1). A comprehensive literature search was performed in PubMed and Scopus from database inception to 29 December 2025 to identify studies reporting survival outcomes and prognostic factors in patients with TBSCC. The search strategy combined terms related to the anatomical site (“external auditory canal”, “external auditory meatus”, “ear canal”, “temporal bone”, “middle ear”, “mastoid”) and malignancy (“squamous cell carcinoma”, “carcinoma”, “cancer”, “malignant”, “tumor”, “tumour”, “SCC”). No restrictions on publication date were applied. The complete database-specific search strategies, including the exact search syntax used for PubMed and Scopus, are provided in Supplementary Table S1. The reference lists of eligible articles were manually screened to identify additional relevant studies. After duplicate removal, two reviewers (A.F. and M.S.) independently screened titles and abstracts for eligibility. Full texts of potentially relevant studies were subsequently assessed according to the predefined inclusion and exclusion criteria. Any disagreement during study selection was resolved by a third reviewer (L.C.). The review protocol was not prospectively registered. The completed PRISMA 2020 checklist is provided as Supplementary Table S2.

### 2.2. Eligibility Criteria

Studies were considered eligible if they met the following inclusion criteria: (i) histopathological diagnosis of squamous cell carcinoma (SCC) of the external auditory canal (EAC) and/or temporal bone (TB); (ii) tumor staging according to the modified Pittsburgh classification; (iii) patients treated with any therapeutic modality, including surgery with or without adjuvant treatment, definitive radiotherapy, or chemoradiotherapy; and (iv) availability of survival analyses reporting hazard ratios (HRs) and corresponding 95% confidence intervals (95% CIs) for tumor stage, nodal status, or surgical margin status. Studies were excluded if they: (i) mixed histological cohorts with non-separable SCC data; (ii) evaluated tumors originating outside the EAC or temporal bone; (iii) represented duplicate publications or overlapping patient cohorts; (iv) lacked relevant prognostic or survival data; (v) were non-original articles, including reviews, editorials, letters, conference abstracts, and meta-analyses; or (vi) were not published in English.

Potential overlap between study populations was assessed by comparing the recruiting institution, study period, sample size, authorship, and reported patient characteristics across publications originating from the same centers or research groups. When potential overlap was suspected, the full texts were cross-checked to determine whether the study populations were independent.

### 2.3. Data Extraction and Risk of Bias Assessment

Data from the included studies were independently extracted by two reviewers (A.F. and M.S.) using a standardized electronic database. Disagreements were resolved by consensus. The following variables were extracted: first author, year of publication, country of origin, study design, sample size, patient demographics, treatment modality, tumor stage, nodal status, surgical margin status, adjuvant treatment, survival outcomes, and hazard ratios (HRs) with corresponding 95% confidence intervals (95% CIs). For each study, HRs evaluating the prognostic impact of tumor stage (T1–2 vs. T3–4), nodal status (N0 vs. N+), and surgical margin status (R0 vs. R1) were extracted. When both univariable and multivariable estimates were available, multivariable HRs were preferentially extracted; when multivariable analyses were unavailable, univariable HRs were used. Because adjusted and unadjusted estimates may differ in their susceptibility to confounding, the type of HR extracted from each study was recorded and is reported in Supplementary Table S3.

The methodological quality of the included studies was assessed using the Newcastle–Ottawa Scale (NOS) [9] for cohort studies. Quality assessment was independently performed by two reviewers (M.S. and L.C.), with disagreements resolved by consensus.

### 2.4. Statistical Analysis

For each prognostic variable of interest, hazard ratios (HRs) and corresponding 95% confidence intervals (95% CIs) were pooled using the inverse-variance method. Random-effects models were used as the primary analytical approach owing to the expected clinical and methodological heterogeneity among the included studies. Hazard ratios greater than 1 indicated poorer overall survival in the exposed group.

Statistical heterogeneity was assessed using Cochran’s Q test and quantified using the I^2^ statistic [10]. Heterogeneity was interpreted according to Higgins et al. as low (0–40%), moderate (30–60%), substantial (50–90%), and considerable (75–100%) [11]. A two-sided *p*-value < 0.05 was considered statistically significant.

Sensitivity analyses were performed using a leave-one-out approach, whereby each study was sequentially removed and the pooled estimate recalculated to evaluate the robustness of the findings. Small-study effects were explored through visual inspection of funnel plots and Egger’s regression test. Given the limited number of studies contributing to some meta-analyses, particularly those including fewer than 10 studies, the results of funnel-plot asymmetry tests were interpreted cautiously. All statistical analyses were performed using R (version 4.3.2; R Foundation for Statistical Computing, Vienna, Austria) [12].

## 3. Results

### 3.1. Study Selection

The literature search identified 2302 records through PubMed (*n* = 986) and Scopus (n = 1316). After duplicate removal, 1458 records underwent title and abstract screening, resulting in the exclusion of 1378 records. Eighty full-text articles were assessed for eligibility, of which six could not be retrieved. Following full-text review, 61 studies were excluded because of insufficient or aggregate outcome data, use of non-modified Pittsburgh staging systems, or publication in languages other than English. Ultimately, 13 retrospective cohort studies met the eligibility criteria and were included in both the qualitative and quantitative synthesis [13,14,15,16,17,18,19,20,21,22,23,24,25] (Figure 1).

### 3.2. Study Characteristics

The 13 included retrospective cohort studies comprised 801 patients with temporal bone squamous cell carcinoma, including 443 males (55.3%) and 358 females (44.7%). Studies were published between 2014 and 2025 and originated predominantly from East Asia, with two additional cohorts from Europe (Italy and the United Kingdom). Sample sizes ranged from 21 to 173 patients. The reported mean or median age ranged from 55.2 to 67.5 years. Follow-up duration, reported in 11 studies, ranged from 14.5 to 74.7 months. Surgery was the primary treatment modality in all included studies, although the extent of temporal bone resection, neck dissection, and parotidectomy varied considerably among cohorts. Table 1 summarizes the main clinicopathological variables collected across the included studies, including modified Pittsburgh T stage, cervical nodal status, surgical margin status, preoperative facial paralysis, prior radiotherapy, treatment characteristics, and survival outcomes.

Newcastle–Ottawa Scale scores ranged from 6 to 8 across the included studies, with the domain-level assessment reported in Table 2 [9].

Sufficient data were available to perform quantitative synthesis for surgical margin status, tumor stage, and cervical nodal status.

### 3.3. Prognostic Impact of Surgical Margin Status

**Figure 2:**
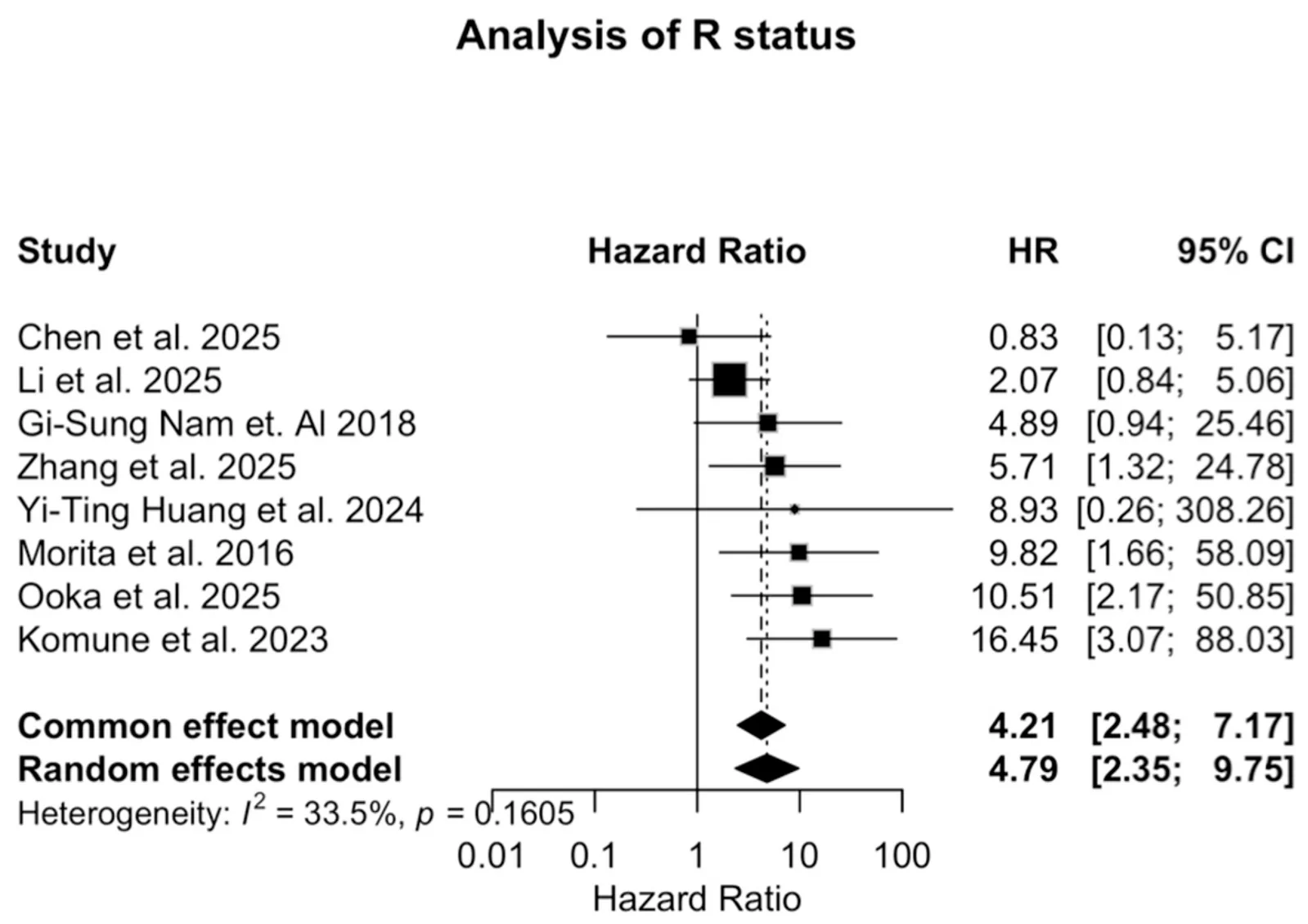
Meta-analysis evaluating surgical margin status [13,14,17,20,21,22,23,24].

**Figure A1:**
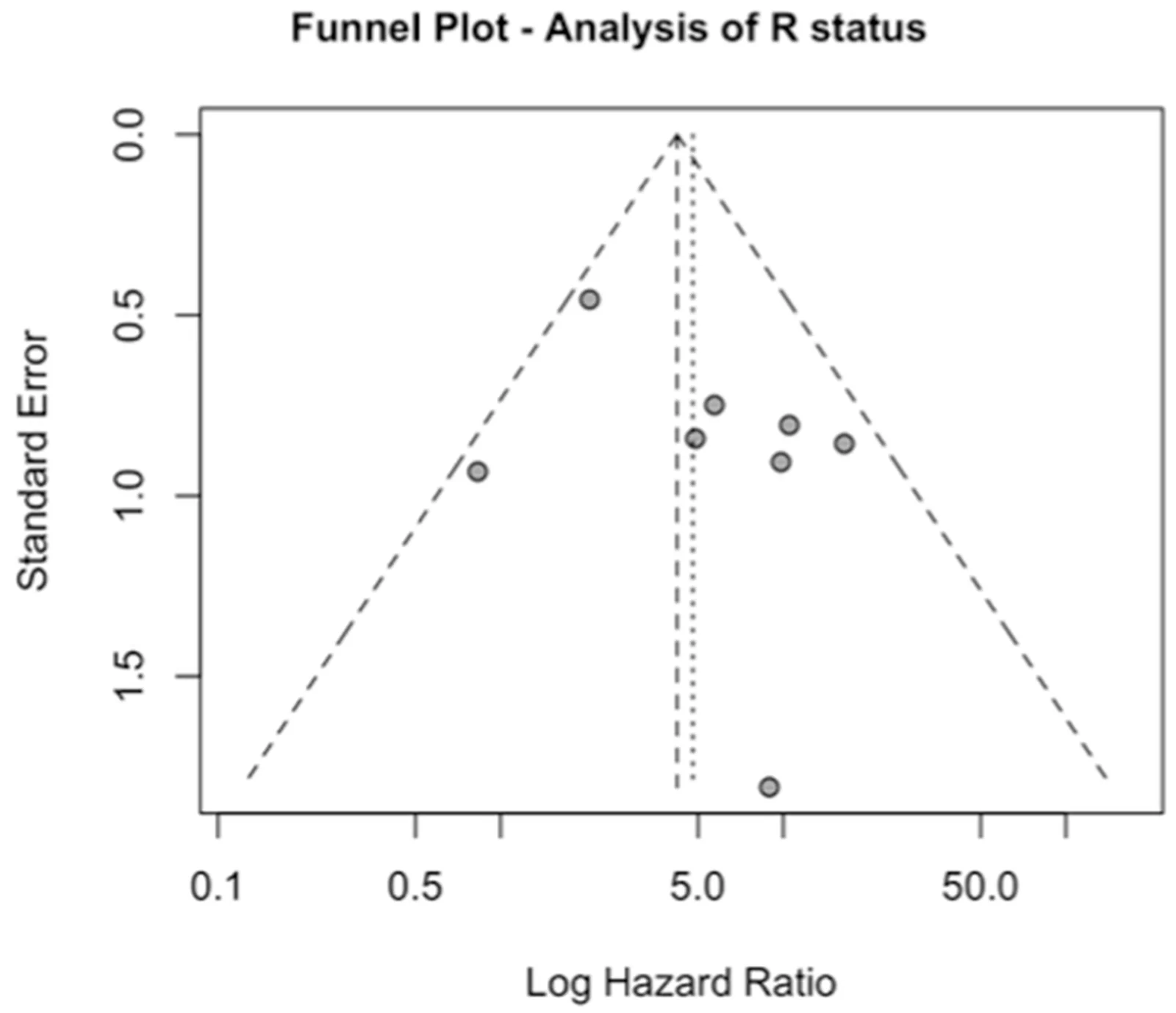
Egger’s regression test of surgical margin status [13,14,17,20,21,22,23,24].

**Figure A2:**
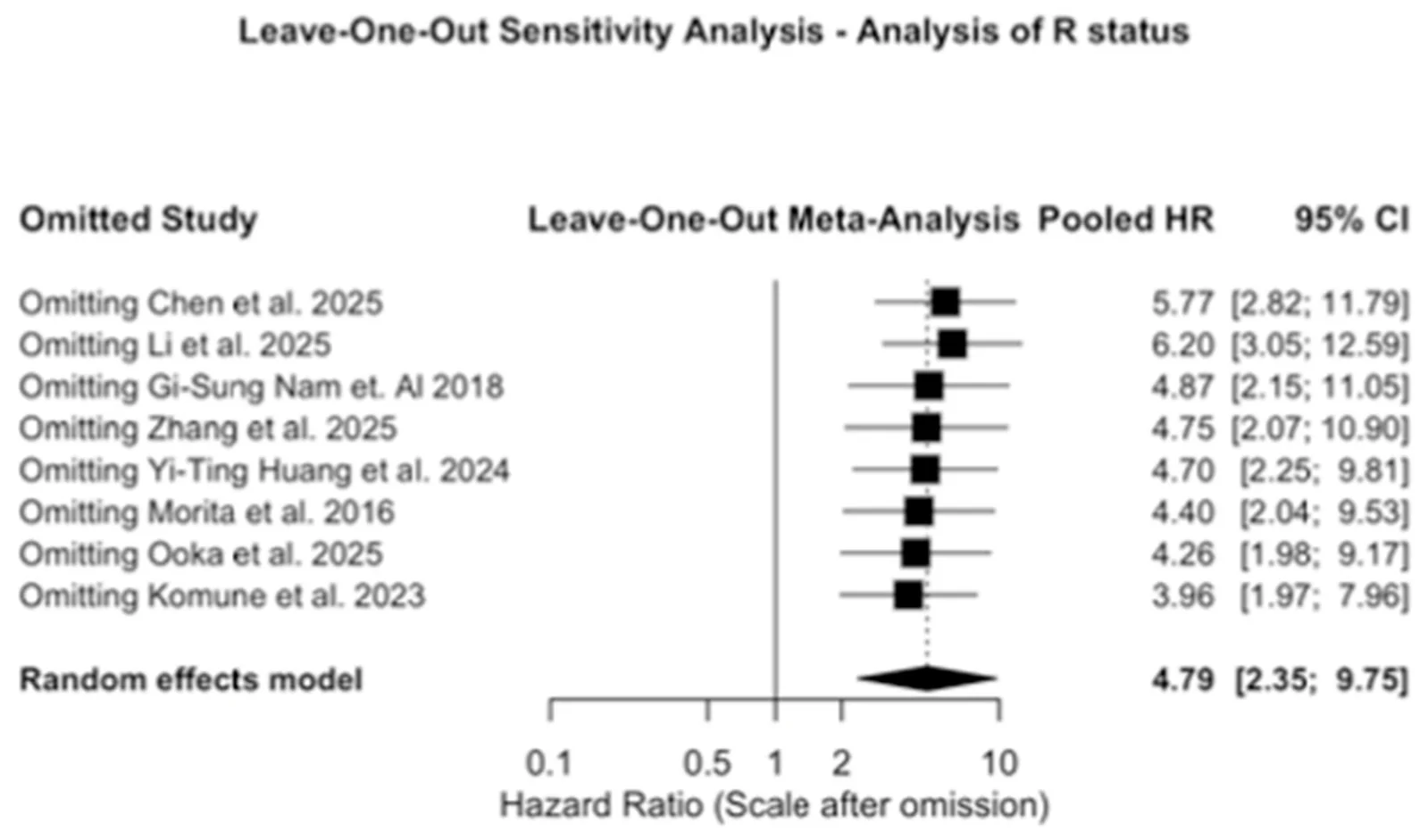
Leave-one-out sensitivity analysis of surgical margin status [13,14,17,20,21,22,23,24].

Eight studies including 540 patients were included in the meta-analysis evaluating surgical margin status (Figure 2). Positive surgical margins were associated with a significantly increased hazard of mortality (pooled HR 4.79, 95% CI, 2.35–9.75; *p* < 0.001). Between-study heterogeneity was low to moderate (I^2^ = 33.5%). Leave-one-out sensitivity analysis demonstrated that no individual study substantially influenced the pooled estimate, confirming the robustness of the pooled estimate. Egger’s regression test did not detect significant funnel-plot asymmetry (*p* = 0.258); however, given the limited number of studies included in this analysis (n = 8), this finding should be interpreted cautiously. (Appendix Figure A1 and Figure A2).

### 3.4. Prognostic Impact of Tumor Stage

**Figure 3:**
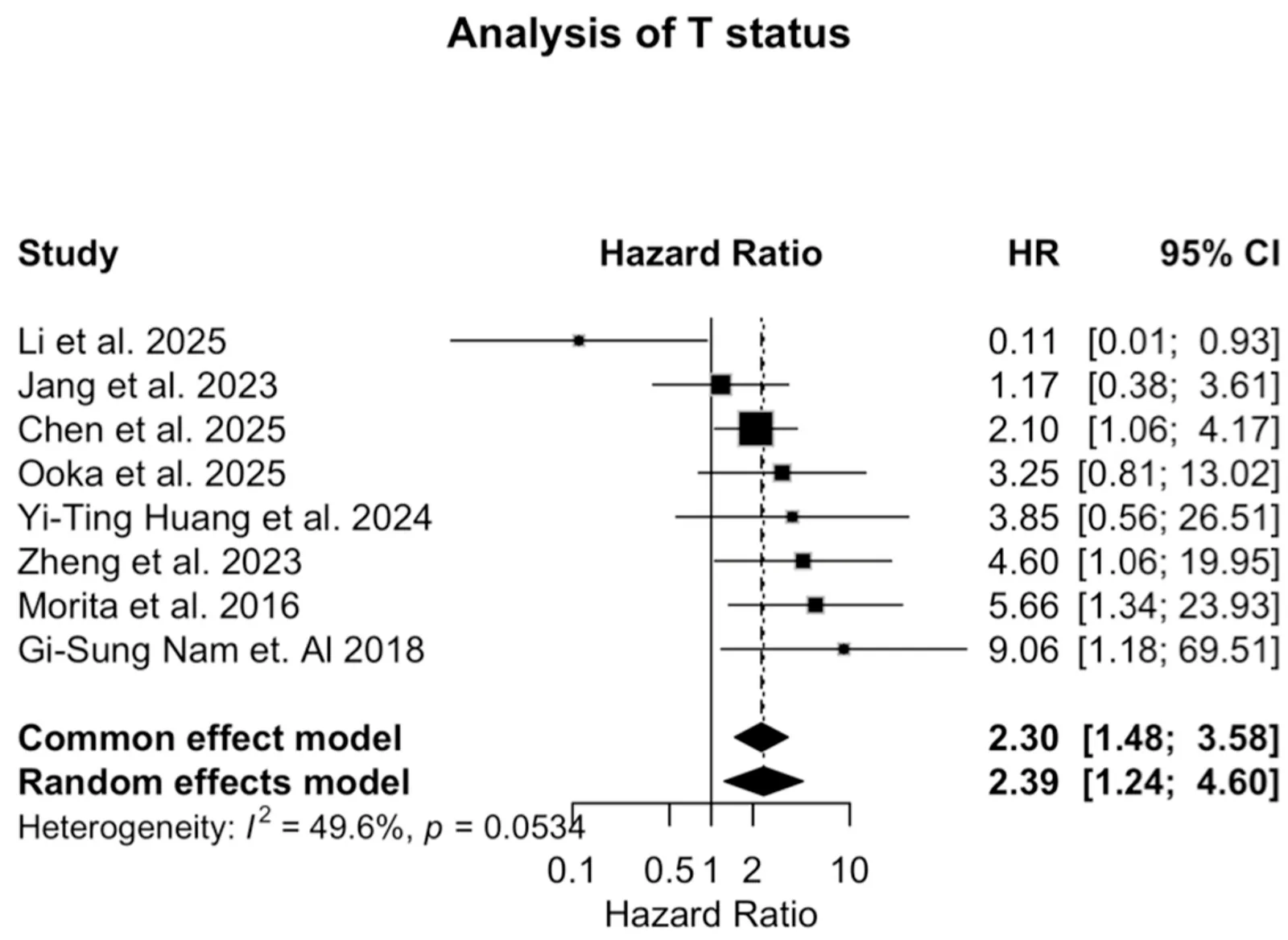
Meta-analysis evaluating tumor stage [13,14,15,20,21,22,23,25].

**Figure A3:**
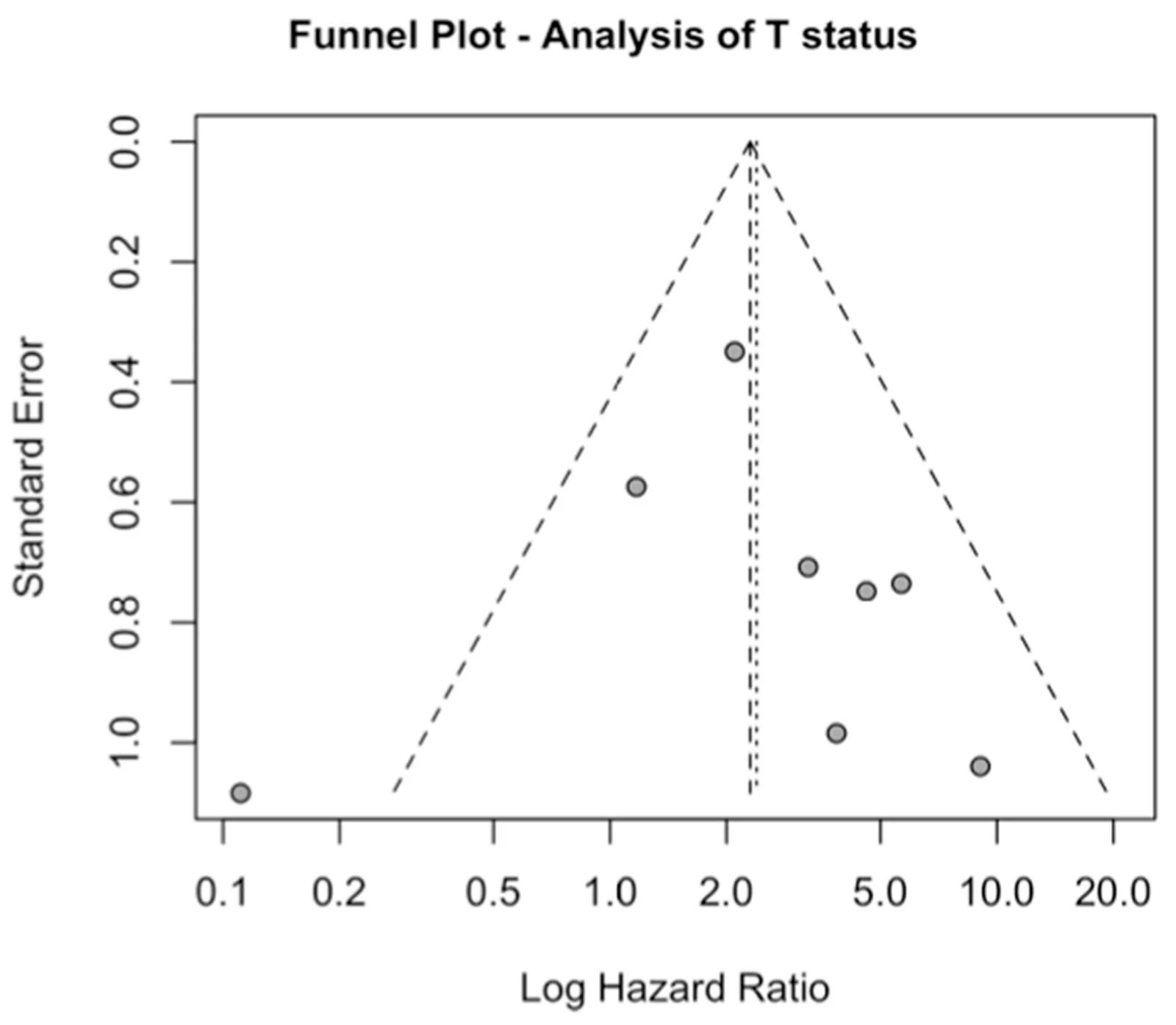
Egger’s regression test of tumor stage [13,14,15,20,21,22,23,25].

**Figure A4:**
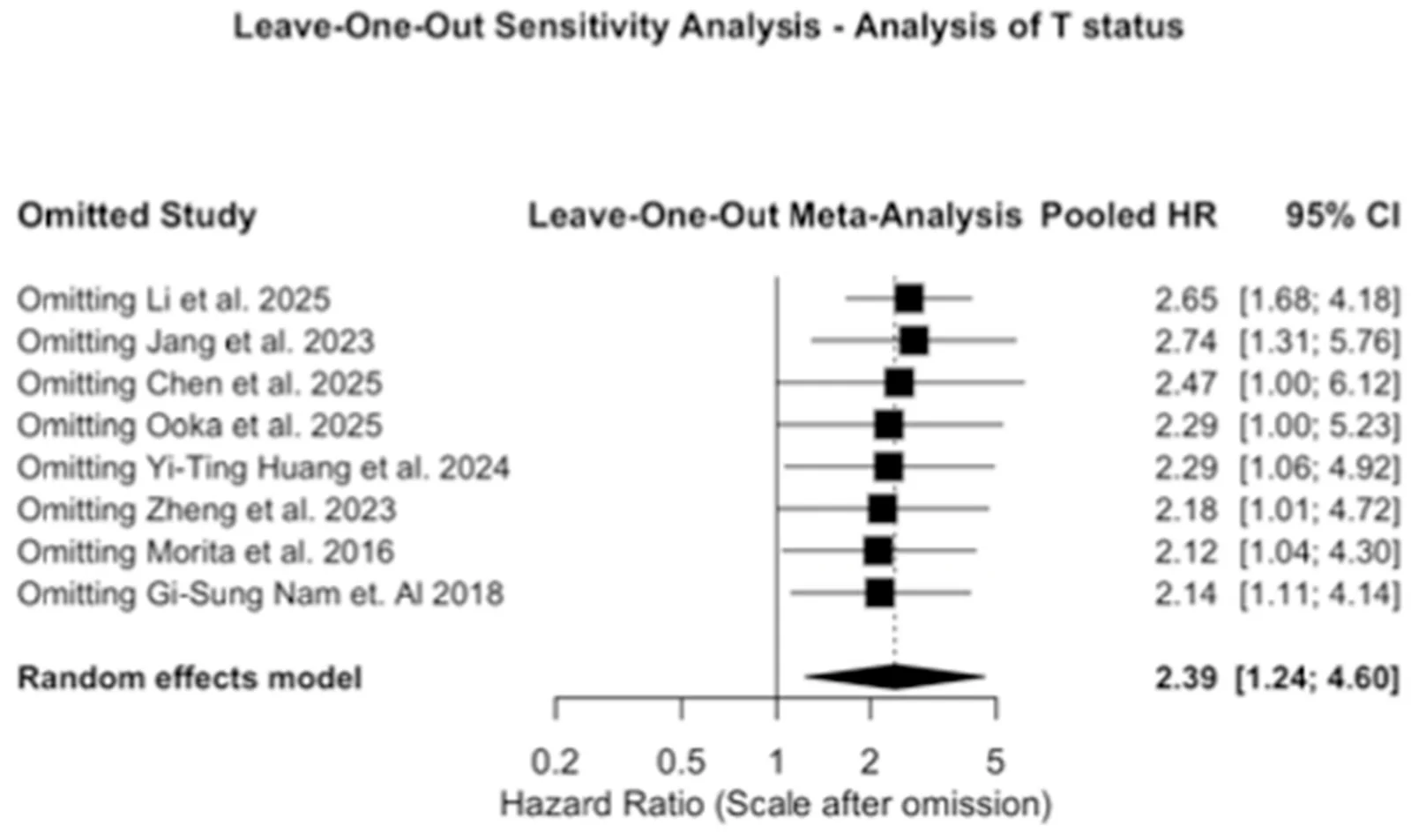
Leave-one-out sensitivity analysis of tumor stage [13,14,15,20,21,22,23,25].

Eight studies including 412 patients reported hazard ratios according to tumor stage (Figure 3). Advanced tumor stage (T3–T4) was associated with significantly poorer overall survival than early-stage tumors (T1–T2), with a pooled HR of 2.39 (95% CI, 1.24–4.60; *p* = 0.009). Moderate between-study heterogeneity was observed (I^2^ = 49.6%). Sensitivity analyses demonstrated stable results following sequential exclusion of individual studies. Egger’s regression test did not detect significant funnel-plot asymmetry (*p* = 0.889); however, the limited number of included studies (n = 8) precludes reliable assessment of small-study effects. (Appendix Figure A3 and Figure A4).

### 3.5. Prognostic Impact of Nodal Status

**Figure 4:**
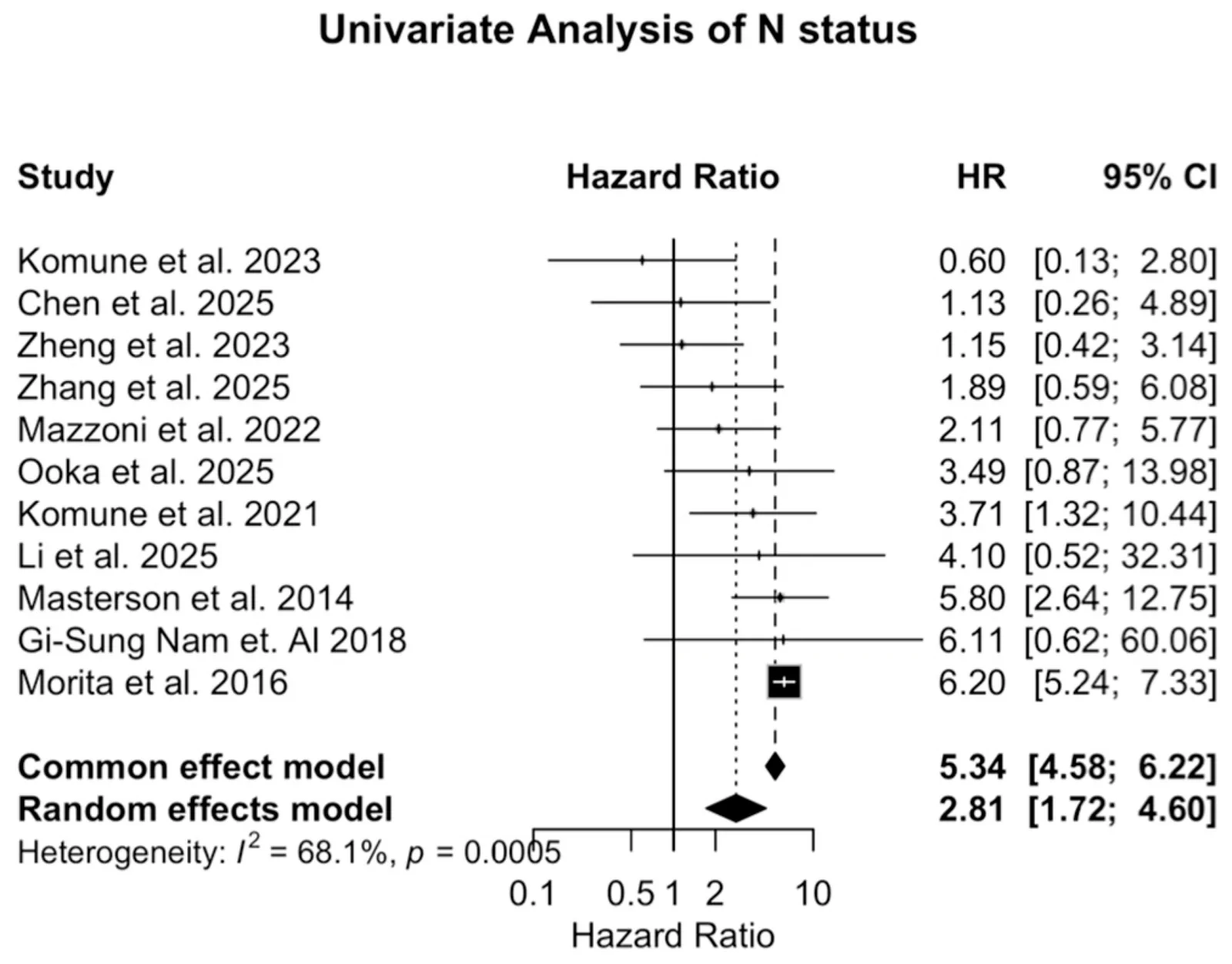
Meta-analysis evaluating nodal status [13,14,16,17,18,19,20,21,23,24,25].

**Figure A5:**
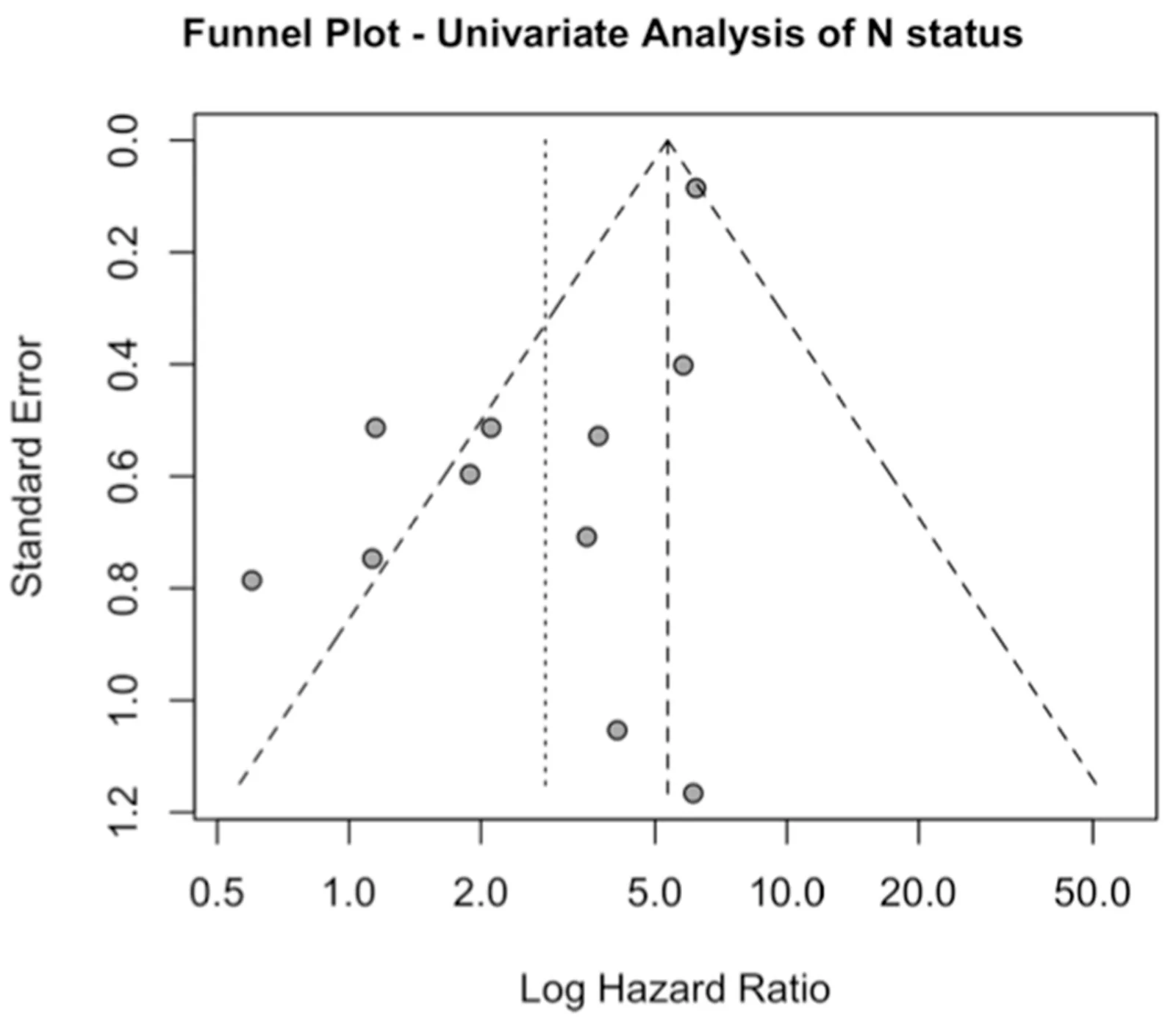
Egger’s regression test of nodal status [13,14,16,17,18,19,20,21,23,24,25].

**Figure A6:**
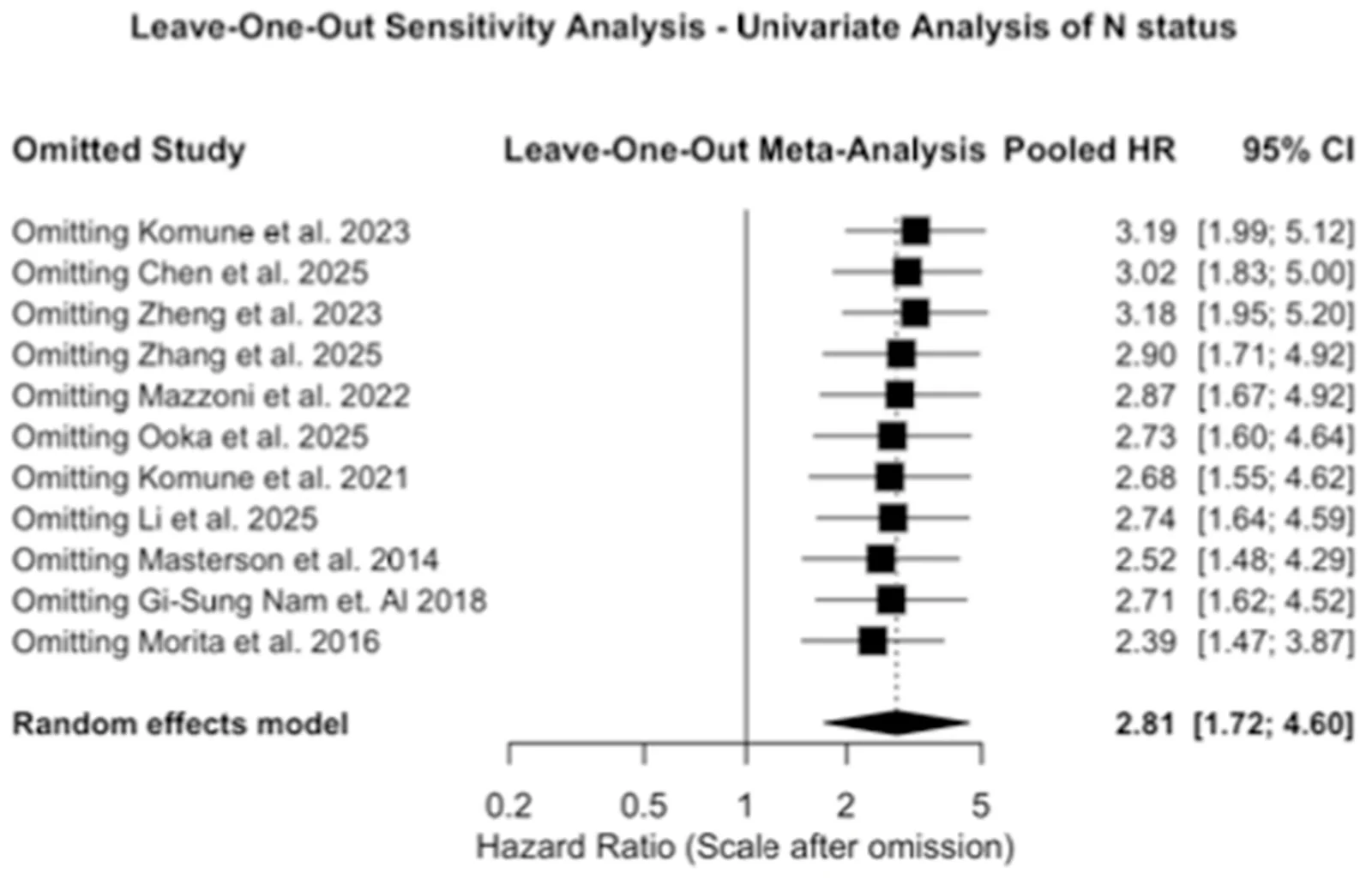
Leave-one-out sensitivity analysis of nodal status [13,14,16,17,18,19,20,21,23,24,25].

Eleven studies including 729 patients investigated the prognostic significance of cervical nodal status (Figure 4). Patients with cervical nodal metastases experienced significantly poorer overall survival than node-negative patients, with a pooled HR of 2.81 (95% CI, 1.72–4.60; *p* < 0.001). Between-study heterogeneity was substantial (I^2^ = 68.1%). The pooled estimate remained stable in leave-one-out sensitivity analyses. Egger’s regression test indicated significant funnel-plot asymmetry (*p* = 0.006), suggesting the presence of small-study effects (Appendix Figure A5 and Figure A6).

## 4. Discussion

To our knowledge, this systematic review and meta-analysis provides the first quantitative synthesis of the principal prognostic determinants of overall survival in TBSCC using pooled hazard ratios. Three factors were consistently associated with worse survival: positive surgical margins, advanced tumor stage, and cervical nodal metastasis. Among these, surgical margin status demonstrated the largest pooled association with mortality, emphasizing the clinical importance of achieving complete oncologic resection whenever technically feasible.

The present findings should also be considered in relation to the previous systematic review and meta-analysis by Cazzador et al. [6]. That study demonstrated an adverse prognostic impact of advanced T category and evaluated nodal status using pooled survival data and study-level odds ratios. The present analysis complements these findings by incorporating more recent evidence through December 2025 and specifically synthesizing study-level time-to-event estimates as hazard ratios for surgical margin status, tumor stage, and cervical nodal involvement. In particular, the quantitative synthesis of surgical margin status provides additional evidence regarding a clinically relevant prognostic factor that could not be meta-analyzed in the previous study because of limited available data.

This finding is consistent with previous studies demonstrating that complete oncologic resection remains the cornerstone of treatment for TBSCC. In particular, Komune et al. reported significantly poorer overall and disease-free survival in patients with positive surgical margins, with postoperative recurrence occurring in seven of nine patients despite the frequent use of adjuvant chemoradiotherapy [17]. Positive surgical margins likely reflect two complementary mechanisms. First, they indicate residual microscopic disease, which may persist despite adjuvant treatment and ultimately contribute to local recurrence. Second, they frequently represent a surrogate marker of technically unresectable or biologically more aggressive tumors involving critical structures such as the carotid canal, dura mater, petrous apex, or facial nerve [5]. Consequently, the adverse prognostic impact of positive margins probably reflects both residual disease and intrinsic tumor aggressiveness. Mazzoni et al. showed that local recurrence remains the predominant pattern of treatment failure following surgery for advanced TBSCC, emphasizing that incomplete local disease control continues to represent the major obstacle to long-term survival [19]. Achieving negative margins in TBSCC often requires careful preoperative imaging, appropriate selection of the extent of temporal bone resection, and multidisciplinary surgical planning. Therefore, surgical margin status should not be regarded solely as a pathological endpoint, but also as an indicator of the adequacy of surgical strategy.

Nevertheless, across the included studies, nearly all patients with positive surgical margins received postoperative radiotherapy, frequently combined with concurrent chemotherapy. Despite the widespread use of adjuvant treatment in this high-risk subgroup, positive surgical margins remained strongly associated with poorer overall survival, suggesting that postoperative radiotherapy may not fully compensate for the adverse prognostic impact of residual disease. This observation is consistent with previous institutional series reporting inferior outcomes despite aggressive multimodal treatment. Moffat et al. observed substantially better disease-free survival in patients undergoing primary radical surgery followed by postoperative radiotherapy than in those treated with salvage surgery (66% vs. 33%), emphasizing the importance of achieving complete resection at the initial surgery [4]. Similar conclusions have been reported in more recent surgical series evaluating multimodal treatment strategies [18,20].

These findings should, however, be interpreted with caution. The marked variability in indications for radiotherapy, radiation protocols, doses, and the use of concurrent chemotherapy precludes any definitive conclusions regarding the independent contribution of adjuvant treatment. Consequently, the observed association between positive margins and poorer survival should not be interpreted as evidence against the value of postoperative radiotherapy but rather reinforces the importance of achieving complete surgical resection whenever technically feasible, with adjuvant therapy serving as a complementary rather than compensatory treatment modality.

Advanced tumor stage also demonstrated a significant association with overall survival, approximately doubling the risk of mortality compared with early-stage disease. This finding is consistent with previous evidence identifying tumor stage as the most established prognostic determinant in TBSCC [3,6,7]. The modified Pittsburgh classification remains clinically valuable because it captures progressive invasion of critical anatomical structures, including the facial nerve, temporomandibular joint, dura mater, carotid canal, and skull base. Increasing T stage therefore reflects not only greater tumor burden but also decreasing likelihood of achieving complete oncologic resection and so increasing the likelihood of locoregional recurrence [5].

Positive cervical nodal status was also associated with significantly poorer overall survival, with an approximately threefold higher hazard of mortality. Cervical nodal metastasis is generally considered a marker of biologically aggressive disease, reflecting increased invasive capacity and greater metastatic potential. Previous institutional series consistently reported cervical nodal metastasis as an adverse prognostic factor in TBSCC [14,15,25]. However, this result should be interpreted cautiously, since substantial statistical heterogeneity was observed across studies and evidence of small-study effects was detected. Nodal metastases remain relatively uncommon in TBSCC, and available studies are limited by small event numbers, which may partially explain the observed heterogeneity and evidence of small-study effects. In addition, differences across cohorts in tumor stage distribution, neck-management strategies, treatment approaches, geographic setting, and treatment era may have contributed to the observed heterogeneity, although inconsistent reporting of these variables precluded a meaningful exploration of their potential impact. Finally, only univariable analyses were available, limiting the ability to establish cervical nodal involvement as an independent prognostic factor.

The present study has several strengths. This is the first meta-analysis pooling hazard ratios. The application of strict eligibility criteria, including only studies using the modified Pittsburgh classification, improved the comparability of the included cohorts. Furthermore, multivariable hazard ratios were preferentially extracted whenever available, and the robustness of the pooled estimates was confirmed through sensitivity analyses.

Several limitations should be acknowledged. First, all included studies were retrospective observational cohorts, making the pooled estimates susceptible to selection bias, residual confounding, and inherent limitations in causal inference. Second, given the rarity of TBSCC, the available evidence was based on relatively small patient cohorts, and substantial heterogeneity existed across studies regarding surgical techniques, indications for parotidectomy and neck dissection, as well as the use of adjuvant radiotherapy and concurrent chemotherapy. Importantly, treatment selection was closely related to disease characteristics, including tumor stage, margin status, and nodal involvement, and may therefore have contributed to confounding of the observed prognostic associations. Furthermore, although multivariable HRs were preferentially extracted, several contributing studies reported only univariable estimates. Because adjusted and unadjusted HRs are not fully equivalent and may differ in their susceptibility to confounding, this represents an important source of methodological heterogeneity and limits interpretation of the pooled estimates as independent prognostic effects. The restriction to English-language publications may also have introduced language bias, particularly given the substantial contribution of East Asian centers to the TBSCC literature. Taken together, these methodological limitations reduce the certainty of the available evidence and warrant cautious interpretation of the pooled estimates, particularly for cervical nodal status.

Overall, our findings suggest that while tumor stage remains a fundamental determinant of prognosis, achieving negative surgical margins represents an important factor associated with survival after surgery for TBSCC, although margin status is closely influenced by disease extent and surgical resectability.

## 5. Conclusions

This systematic review and meta-analysis provides the first quantitative synthesis of the principal prognostic factors associated with overall survival in temporal bone squamous cell carcinoma. Positive surgical margins, advanced tumor stage, and cervical nodal metastasis were all significantly associated with poorer survival, with positive surgical margins showing the largest pooled association with mortality among the three evaluated factors. Although the available evidence is limited by the retrospective design and heterogeneity of published studies, this analysis provides more robust estimates of the relative prognostic impact of these variables and may support surgical planning, patient counselling, and risk stratification. Future prospective multicenter studies using standardized staging and treatment protocols are needed to validate these findings.

## Figures and Tables

**Table 1:** Included study characteristics [13,14,15,16,17,18,19,20,21,22,23,24,25]. Abbreviations: NR, not reported; RT, radiotherapy; CT, chemotherapy; SR, sleeve resection; LCR, local canal resection; LTBR, lateral temporal bone resection; ELTBR, extended lateral temporal bone resection; STBR, subtotal temporal bone resection; TTBR, total temporal bone resection; ETBR, extended temporal bone resection; R0, negative surgical margins; R1, positive surgical margins; FU, follow-up; N0, absence of cervical nodal metastasis; N+, presence of cervical nodal metastasis.

Author	Year	Type of Study	Country	N. of Patients (Male)	Age (Years)	Facial Palsy	Prior RT	T Stage	N Stage	Surgery	Neck Dissection	Parotidectomy	R Status	Adjuvant RT	FU (Months)
Ooka et al. [21]	2025	Retrospective	Japan	73 (37)	65 (40–93)	NR	0	T1 32; T2 5; T3 14; T4 14	N0 61; N+ 12	LTBR 48, STBR 11, Partial resection 2	-	22	R0 44; R1 17	43 RT; 12 of which with concomitant CT	23.8 (2.8–93.6)
Zhang et al. [24]	2025	Retrospective	China	173 (102)	55.2 ± 14.36	NR	26	T1–3 46; T4 127	N0 156; N+ 17	LCR 10; LTBR 87; STBR 75; TTBR 1	77	105	R0 49; R1 124	147 RT, 47 of which with concomitant CT	67 (2–141)
Chen et al. [13]	2025	Retrospective	China	81 (47)	66 ± 11	11	0	T1 18; T2 17; T3 14; T4 32	N0 75; N+ 6	-	-	20	R0 22; R1 20	-	24.2 (3–234)
Li et al. [23]	2024	Retrospective	China	44 (27)	65 (36–89)	NR	0	T1 10, T2 5; T3 18; T4 11	N0 43; N+ 1	LTBR 24; STBR 20	3	6	R0 28; R1 16	29 RT, of which 4 with concomitant CT	33 (6–103)
Yi-Ting Huang et al. [22]	2024	Retrospective	Taiwan	21 (13)	61 ± 9.9	3	3	T1 5; T2 3; T3 4; T4 9	N0 21	SR 8; LTBR 9; STBR 1; Partial resection 2	7	4	R0 10; R1 8	13 RT, of which 9 with concomitant CT	52.2 (2.3–149.5)
Komune et al. [17]	2023	Retrospective	Japan	40 (15)	67.5 (33–83)	1	2	T1 1; T2 16; T3 11; T4 12	N0 29; N+ 11	LTBR 34; STBR 6	-	-	R0 31; R1 9	6	25.3 (6.5–64.3)
Jang et al [15].	2023	Retrospective	Singapore	51 (34)	64.3 ± 11.6	10	9	T1 11; T2 8; T3 11 T4 21	N0 44; N+ 7	SR 5; LTBR 28; STBR 8	18	24	R0 38; R1 3	26, 2 of which with CT	74.7 (6–297)
Zheng et al. [25]	2023	Retrospective	China	50 (38)	61 (30–79)	6	0	T1–2 7; T3 17; T4 20	N0 36; N+ 8	-	-	-	-	14	-
Mazzoni et al. [19]	2022	Retrospective	Italy	45 (20)	60 (37–82)	8	0	T1 7; T2 7; T3 8; T4 23	N0 29; N+ 9	LTBR 31; STBR 14	38	43	-	26	58 (1–232)
Komune et al. [17]	2021	Retrospective	Japan	71 (28)	66 (37–88)	NR	0	T1 12; T2 17; T3 17; T4 25	N0 58; N+ 13	SR 4; LTBR 43; STBR 4	27	22	R0 34; R1 11	8	-
Gi-Sung Nam et al. [14]	2018	Retrospective	South Korea	26 (16)	63 (40–72)	3	1	T1 11; T2 6; T3 4; T4 5	N0 23; N+ 3	SR 3; LTBR 17; ELTBR 4; STBR: 2	12	20	R0 19; R1 7	12	36.3 (2.2–98.6)
Morita et al. [20]	2016	Retrospective	Japan	66 (35)	65 (29–88)	-	0	T1 24; T2 14; T3 14; T4 14	N0 54; N+ 12	LTBR 31; STBR 3	12	13	R0 18; R1 16	17, 2 of which with CT	37.5 (6–120)
Masterson et al. [18]	2014	Retrospective	UK	60 (31)	62.5 (37–80)	-	3	T2 8; T3 9; T4 43	N0 49; N+ 11	LTBR 12; ETBR 48	60	60	R0 26; R1 15	40	14.5 (1–216)

**Table 2:** Risk of bias assessment according to Newcastle–Ottawa scale [13,14,15,16,17,18,19,20,21,22,23,24,25].

Study	S1	S2	S3	S4	C	O1	O2	O3	Total
Masterson 2014 [18]	1	0	1	1	2	1	1	1	8
Morita 2016 [20]	1	0	1	1	2	1	1	1	8
Nam 2018 [14]	1	0	1	1	0	1	1	1	6
Komune 2021 [16]	1	0	1	1	0	1	1	1	6
Mazzoni 2022 [19]	1	0	1	1	1	1	1	1	7
Jang 2023 [15]	1	0	1	1	2	1	1	1	8
Komune 2023 [17]	1	0	1	1	0	1	1	1	6
Zheng 2023 [25]	1	0	1	1	1	1	1	1	7
Huang 2024 [22]	1	0	1	1	0	1	1	1	6
Chen 2025 [13]	1	0	1	1	2	1	1	1	8
Ooka 2025 [21]	1	0	1	1	1	1	1	1	7
Wei Li 2025 [23]	1	0	1	1	2	1	1	1	8
Zhang 2025 [24]	1	0	1	1	0	1	1	1	6

## Data Availability

All data analyzed in this study were derived from previously published studies. The data supporting the findings of this study are available from the corresponding author upon reasonable request.

## Supplementary Materials

The following supporting information can be downloaded at: https://www.mdpi.com/article/10.3390/jcm15187143/s1, Table S1: Complete database-specific search strategies; Table S2: PRISMA 2020 checklist; Table S3: HRs extracted from individual studies.

## Abbreviations

The following abbreviations are used in this manuscript:

TBSCC	Temporal Bone Squamous Cell Carcinoma
EAC	External Auditory Canal
