# Supplementary material for: Survival Determinants in Temporal Bone Squamous Cell Carcinoma: A Systematic Review and Meta-Analysis
Source: J Clin Med. 2026 Sep 15;15(18):7143. doi: 10.3390/jcm15187143 (PMC13607753; doi:10.3390/jcm15187143)
Supplement: Supplementary file 1 [file jcm-15-07143-s001.zip › Tables S1 and S3.pdf]

Supplementary Table S1. Complete database-specific search strategies.

Database	Date of final search	Complete search strategy	Records retrieved
PubMed	29 December 2025	("temporal bone" OR "temporal bones" OR "external auditory canal" OR "external auditory canals" OR "external auditory meatus" OR "external acoustic meatus" OR "ear canal" OR "ear canals" OR "auditory canal" OR "auditory canals" OR "middle ear" OR "petrous bone" OR "petrous apex" OR "mastoid")	986
Scopus	29 December 2025	TITLE-ABS-KEY (("temporal bone" OR "temporal bones" OR "external auditory canal" OR "external auditory canals" OR "external auditory meatus" OR "external acoustic meatus" OR "auditory canal" OR "auditory canals" OR "ear canal" OR "ear canals" OR "middle ear" OR "petrous bone" OR "petrous apex" OR "mastoid") AND ("squamous cell carcinoma" OR "squamous cell carcinomas" OR "squamous cell cancer" OR "squamous cell cancers" OR "squamous cell neoplasm" OR "squamous cell neoplasms" OR "squamous carcinoma" OR "squamous carcinomas" OR "SCC" OR "TBSCC" OR "EACSCC"))	1316

Supplementary Table S2. PRISMA 2020 checklist

Supplementary Table S3. HRs extracted from individual studies.

Study	Margin HR (95% CI)	Analysis	T-stage HR (95% CI)	Analysis	N-status HR (95% CI)
Masterson 2014	/	/	/	/	5,800 (2,400 - 11,600)
Morita 2016	9,820 (1,660 - 58,100)	Univariate	5,660 (1,510 - 27,000)	Multivariate	6,200 (5,050 - 7,060)
Nam 2018	4,890 (0,940 - 25,480)	Univariate	/	/	6,110 (0,620 - 59,920)
Komune 2021	/	/	/	/	3,710 (1,320 - 10,460)
Mazzoni 2022	/	/	/	/	2,110 (0,770 - 5,760)
Jang 2023	/	/	1,170 (0,380 - 3,610)	Multivariate	/
Komune 2023	16,450 (3,900 - 111,690)	Univariate	/	/	0,600 (0,130 - 2,830)
Zheng 2023	/	/	4,600 (1,060 - 19,930)	Univariate	1,150 (0,420 - 3,140)
Huang 2024	8,930 (0,990 - 1.179,840)	Univariate	3,850 (0,790 - 37,470)	Univariate	/
Chen 2025	0,830 (0,150 - 5,820)	Univariate	2,100 (1,070 - 4,210)	Multivariate	1,130 (0,260 - 4,860)
Ooka 2025	10,510 (2,170 - 50,800)	Univariate	3,250 (0,810 - 13,000)	Univariate	3,490 (0,870 - 13,970)
Li 2025	2,066 (0,843 - 5,051)	Multivariate	0,111 (0,013 - 0,911)	Multivariate	4,098 (0,519 - 32,258)
Zhang 2025	5,711 (1,316 - 24,786)	Multivariate	/	/	1,889 (0,587 - 6,076)
